# Dominance of Three Sublineages of the SARS-CoV-2 Delta Variant in Mexico

**DOI:** 10.3390/v14061165

**Published:** 2022-05-27

**Authors:** Blanca Taboada, Selene Zárate, Rodrigo García-López, José Esteban Muñoz-Medina, Alejandro Sanchez-Flores, Alfredo Herrera-Estrella, Celia Boukadida, Bruno Gómez-Gil, Nelly Selem Mojica, Mauricio Rosales-Rivera, Angel Gustavo Salas-Lais, Rosa María Gutiérrez-Ríos, Antonio Loza, Xaira Rivera-Gutierrez, Joel Armando Vazquez-Perez, Margarita Matías-Florentino, Marissa Pérez-García, Santiago Ávila-Ríos, Juan Manuel Hurtado, Carla Ivón Herrera-Nájera, José de Jesús Núñez-Contreras, Brenda Sarquiz-Martínez, Víctor Eduardo García-Arias, María Guadalupe Santiago-Mauricio, Bernardo Martínez-Miguel, Julissa Enciso-Ibarra, Cristóbal Cháidez-Quiróz, Pavel Iša, Rosa María Wong-Chew, María-Eugenia Jiménez-Corona, Susana López, Carlos F. Arias

**Affiliations:** 1 Departamento de Genética del Desarrollo y Fisiología Molecular, Instituto de Biotecnología, Universidad Nacional Autónoma de México, Cuernavaca 62210, Morelos, Mexico; rodrigo.garcia@ibt.unam.mx (R.G.-L.); mauricio.rosales@ibt.unam.mx (M.R.-R.); aloza242@gmail.com (A.L.); xaira.rivera@ibt.unam.mx (X.R.-G.); pavel.isa@ibt.unam.mx (P.I.); susana.lopez@ibt.unam.mx (S.L.); 2 Posgrado en Ciencias Genómicas, Universidad Autónoma de la Ciudad de México, Ciudad de México 03100, Mexico; 3 Coordinación de Calidad de Insumos y Laboratorios Especializados, Instituto Mexicano del Seguro Social, Ciudad de México 07760, Mexico; eban10@hotmail.com; 4 Unidad Universitaria de Secuenciación Masiva y Bioinformática, Instituto de Biotecnología, Universidad Nacional Autónoma de México, Cuernavaca 62210, Morelos, Mexico; alejandro.sanchez@ibt.unam.mx; 5 Centro de Investigación y de Estudios Avanzados del IPN, Laboratorio Nacional de Genómica para la Biodiversidad-Unidad de Genómica Avanzada, Irapuato 36824, Guanajuato, Mexico; alfredo.herrera@cinvestav.mx; 6 Centro de Investigación en Enfermedades Infecciosas, Instituto Nacional de Enfermedades Respiratorias Ismael Cosío Villegas, Ciudad de México 14080, Mexico; celia.boukadida@cieni.org.mx (C.B.); margarita.matias@cieni.org.mx (M.M.-F.); marissa.perez@cieni.org.mx (M.P.-G.); santiago.avila@cieni.org.mx (S.Á.-R.); 7 Centro de Investigación en Alimentación y Desarrollo AC, Mazatlán 82000, Sinaloa, Mexico; bruno@ciad.mx (B.G.-G.); juli.encisoi@gmail.com (J.E.-I.); 8 Centro de Ciencias Matemáticas, Universidad Nacional Autónoma de México, Morelia 58089, Michoacan, Mexico; nselem@matmor.unam.mx; 9 Centro de Investigación en Ciencias, Universidad Autónoma de Estado de Morelos, Cuernavaca 62209, Morelos, Mexico; 10 Laboratorio Central de Epidemiología, Instituto Mexicano del Seguro Social, Ciudad de México 02990, Mexico; salas_lais@yahoo.com.mx (A.G.S.-L.); bsarquiz@gmail.com (B.S.-M.); 11 Departamento de Microbiología Molecular, Instituto de Biotecnología, Universidad Nacional Autónoma de México, Cuernavaca 62210, Morelos, Mexico; rosa.gutierrez@ibt.unam.mx; 12 Instituto Nacional de Enfermedades Respiratorias Ismael Cosío Villegas, Ciudad de México 14080, Mexico; joevazpe@gmail.com; 13 Unidad de Computo, Instituto de Biotecnología, Universidad Nacional Autónoma de México, Cuernavaca 62210, Morelos, Mexico; jmanuel.hurtado@ibt.unam.mx; 14 Instituto Mexicano del Seguro Social, Unidad de Investigación Médica Yucatán, Mérida 97150, Yucatán, Mexico; herrera_carla@hotmail.com; 15 Unidad de Investigación Biomédica de Zacatecas, Instituto Mexicano del Seguro Social, Zacatecas 98000, Mexico; chuy_nc@hotmail.com; 16 Centro de Investigación Biomédica de Occidente, Instituto Mexicano del Seguro Social, Guadalajara 44340, Jalisco, Mexico; victorega85@gmail.com; 17 Centro de Investigación Biomédica del Noreste, Instituto Mexicano del Seguro Social, Monterrey 64720, Nuevo León, Mexico; gpe_mauricio83@yahoo.com; 18 División de Laboratorios Especializados, Instituto Mexicano del Seguro Social, Ciudad de México 07760, Mexico; bernardo.martinezm@imss.gob.mx; 19 Laboratorio Nacional para la Investigación en Inocuidad Alimentaria, Centro de Investigación en Alimentación y Desarrollo AC, Culiacán 80129, Sinaloa, Mexico; chaqui@ciad.mx; 20 Laboratorio de Investigación en Enfermedades Infecciosas, División de investigación, Facultad de Medicina, Universidad Nacional Autónoma de Mexico, Ciudad de México 04510, Mexico; rmwong@unam.mx; 21 Departamento de Epidemiología, Instituto Nacional de Cardiología Ignacio Chávez, Ciudad de México 14080, Mexico; mejimenez777@gmail.com

**Keywords:** SARS-CoV-2, variants of concern, delta variant, genomic surveillance

## Abstract

In this study, we analyzed the sequences of SARS-CoV-2 isolates of the Delta variant in Mexico, which has completely replaced other previously circulating variants in the country due to its transmission advantage. Among all the Delta sublineages that were detected, 81.5 % were classified as AY.20, AY.26, and AY.100. According to publicly available data, these only reached a world prevalence of less than 1%, suggesting a possible Mexican origin. The signature mutations of these sublineages are described herein, and phylogenetic analyses and haplotype networks are used to track their spread across the country. Other frequently detected sublineages include AY.3, AY.62, AY.103, and AY.113. Over time, the main sublineages showed different geographical distributions, with AY.20 predominant in Central Mexico, AY.26 in the North, and AY.100 in the Northwest and South/Southeast. This work describes the circulation, from May to November 2021, of the primary sublineages of the Delta variant associated with the third wave of the COVID-19 pandemic in Mexico and highlights the importance of SARS-CoV-2 genomic surveillance for the timely identification of emerging variants that may impact public health.

## 1. Introduction

Lineage B.1.617.2 of SARS-CoV-2 was first detected in India in October 2020. The World Health Organization (WHO) first classified it as a variant of interest (VOI) on 4 April 2021, and later as a variant of concern (VOC) on 11 May 2021, receiving the denomination of Delta [1]. By May 2021, the global spread of this variant had accelerated and quickly displaced all other circulating lineages throughout the world [2], accounting for 99.7% of SARS-CoV-2 infections by mid-November 2021, until the emergence of Omicron, the VOC that eventually displaced it.

As the Delta lineage diversified, a dynamic classification into sublineages was established using the nomenclature “AY.X”, giving rise to over 129 sublineages within the Pango classification (https://cov-lineages.org accessed on 2 December 2021) [3] distributed across the different geographical regions. Therefore, the Delta variant is comprised of a group of distinct sublineages (AY.1–AY.133) which accumulated specific mutations while circulating regionally or locally. The core mutations that confer Delta its transmissibility advantage in the original B.1.617.2 lineage [4] consist of eighteen nonsynonymous substitutions and two deletions, with seven mutations and one deletion in the spike protein (S). Some of these amino acid (aa) changes have been characterized more thoroughly, such as S:T478K, which confers a higher receptor binding affinity [5]; S:L452R, which can increase transmissibility and immune evasion [6]; and S:P681R, which has been shown to improve fusogenicity [7]. Additionally, sublineages such as AY.4.2 present a higher transmissibility than the parental lineage [8], while some specific mutations, such as S:A222V, may be associated with the slightly higher viral titers thought to result in the higher transmissibility of lineage B.1.177 viruses [9], or, as in the case of S:E484K, may enhance immune evasion [10]. Therefore, genomic surveillance is critical for tracking the evolution and spread of SARS-CoV-2 lineages to understand their transmission and spread and to detect specific mutations linked to changes in virulence and immune evasion, especially in a population with increasingly higher vaccination levels.

In Mexico, SARS-CoV-2 genomic surveillance has enabled the study of the expansion, and eventual succession, of increasingly-transmissible variants, such as B.1.1.519, which became dominant in late 2020 during the second pandemic wave observed in the country, peaking at 81% in March 2021 [11,12]. Later, the VOCs Alpha (B.1.1.7) and Gamma (P.1 and its sublineages) circulated in Mexico, albeit with a different geographical distribution, reporting their highest prevalence in May (20%) and June (41%) 2021, respectively, during the period between the second and third waves. The Delta variant was associated with Mexico’s third wave of infections, starting its spread in May 2021. Delta reached over a 90% prevalence in August 2021 and 99% in September, and remained dominant until the arrival of Omicron in December of the same year.

In this study, we characterized the dynamic distribution of the most prevalent sublineages of Delta in Mexico: AY.3, AY.20, AY.26, AY.62, AY.100, AY.103, and AY.113, from May through November 2021. These sublineages showed different geographic distribution patterns, with some dominating specific regions. Moreover, AY.20, AY.26, and AY.100, which accounted for 81.5 % of the infections during this period, were nearly exclusive to and probably originated in Mexico; their dispersion and mutations are thoroughly studied here.

## 2. Materials and Methods

### 2.1. Samples

Oropharyngeal or nasopharyngeal samples were obtained from all 32 Mexican states as part of the national governmental epidemiological surveillance program. Most samples were collected in public clinics and hospitals of the Ministry of Health of Mexico [Instituto Mexicano del Seguro Social (IMSS) and Instituto Nacional de Enfermedades Respiratorias (INER)]. The RNA from 14,529 samples positive for SARS-CoV-2, as confirmed by real-time reverse transcription-polymerase chain reaction (RT-qPCR) and anonymized before the start of the study, were analyzed by the CoViGen-Mex consortium under the Mexican Official Norm NOM-017-SSA2-2012 [13] between February 2020 and November 2021, as previously described [14]. Sequencing was carried out using the Illumina COVIDSeq kit or Nextera XT (Illumina, Inc., San Diego, CA, USA) following the manufacturer’s instructions, using different Illumina platforms and 2 × 150 cycles of paired-end runs. FASTQ files were generated by the Illumina pipeline at BaseSpace (https://basespace.illumina.com accessed on 8 December 2021). Adapters, low-quality bases, and duplicate reads were removed from each sample with a customized pipeline as previously described [15], and the remaining reads were mapped with Bowtie2 v2.3.4.3 [16] against the Wuhan-Hu-1 (NC_045512) reference genome. Afterward, consensus sequences were generated using iVar (v1.3.1) [17] with the following settings: base score Q > 20, 10× minimum read coverage for basecalling, and a 55% threshold under majority rule for single nucleotide polymorphism (SNP) calling. Lineage assignment was performed using the Pangolin lineage classification software tool [3].

### 2.2. Mexican Delta Sequences Dataset

As of 14 December 2021, the CoViGen-Mex consortium has deposited 13,600 genome sequences with a coverage >90%, along with accompanying metadata, in the Global Initiative on Sharing All Influenza Data (GISAID) platform (https://www.gisaid.org/ accessed on 14 December 2021) [18]. Of these sequences, 6685 were assigned as Delta (B.1.617.2 or AY.x) and deposited in GenBank (Supplementary Table S1). An additional dataset of 13,072 comprised of all the other available Mexican Delta sequences and their metadata was retrieved from GISAID in the same period, summing the 19,757 Delta variant genomes from Mexico used in this study (Supplementary Table S1).

### 2.3. Geographical Circulation of Delta Sublineages

To analyze the variation in the most prevalent Delta sublineages in Mexico, sequences from AY.3 (*n* = 1001), AY.20 (*n* = 9759), AY.26 (*n* = 4676), AY.62 (*n* = 224), AY.100 (*n* = 1650), AY.103 (*n* = 215), and AY.113 (*n* = 572) were specifically studied. Moreover, to inspect their geographical distribution and spread, the country was divided into seven regions (Figure S1) as previously reported [14], each including the following states: Northwest (NW): Baja California, Baja California Sur, Chihuahua, Durango, Sonora, and Sinaloa; Northeast (NE): Coahuila, Nuevo Leon, and Tamaulipas; Central North (CN): Aguascalientes, Guanajuato, Querétaro, San Luis Potosi, and Zacatecas; Central South (CS): Mexico City, State of Mexico, Morelos, Hidalgo, Puebla, and Tlaxcala; West (W): Colima, Jalisco, Michoacan, and Nayarit; South (S): Guerrero, Oaxaca, Chiapas, Veracruz, and Tabasco; Southeast (SE): Campeche, Yucatan, and Quintana Roo. A stack density plot was built for each region using ggplot2 v.3.3.5 [19] in R v.4.1.0. Additionally, correlation analyses were performed to assess the differences in the lineage distributions among regions.

### 2.4. Mutation Description

Only sequences from the three most prevalent Delta sublineages in Mexico, AY.20, AY.26, and AY.100, accounting for 81.5% of the sequenced genomes, were included in the analysis of their mutations and spread over time. Synonymous/nonsynonymous substitutions were identified using the Nextclade single nucleotide variant (SNV) calling system [20]. Only SNVs present in more than 1% of the genomes were considered for downstream analyses. A heatmap of mutation frequency was built using ComplexHeatmap v.2.8.0 [21] in R.

### 2.5. Phylogeny and Haplotype Network

A randomized subset of 1190 sequences, representative of AY.20, AY.26, and AY.100, was evenly drawn from all the Mexican states, reflecting the sublineages’ monthly circulation proportions in Mexico. Additionally, 591 genomic sequences from other countries were included: 295 from the rest of North America, 88 each from Europe and South America, 60 from Asia, and 30 each from Africa and Oceania. These sequences were aligned against the reference Wuhan-Hu-1 isolate sequence (NC_045512) using MAFFTv7 with the option -addfragments [22]. The reference sequence and the UTR regions were manually removed from the alignment. A maximum-likelihood phylogeny was built using iqtree2 [23], with the GTR + R + F3 substitution model [24], and time-scaled using LSD2 and collection dates [25]. Finally, the ggtree v.3.0.2 [26] and treeio v.1.16.1 [27] packages were used to display the tree using R. The alignment was converted into the Nexus format and imported into the PopArt v.1.7 software [28] to generate a Templeton, Crandall, and Sing (TCS) network [29], masking sites with more than 5% undefined states. Each sample’s lineage and location information were used to estimate and visualize the geographical and lineage relationships of haplotype groups. SNVs were associated with specific haplotype groups.

### 2.6. Statistical Analysis

Independence *χ*^2^ tests were carried out in R with 10,000 Monte Carlo replicates to evaluate the association between sublineages and categorical variables [gender, patient status, cycle threshold (Ct, evaluating different binning sizes 1, 2, 5, and 10), region, and age]. The results were evaluated with pairwise student’s t-tests with 10,000 resamples, using the lowest sublineage depth to overcome sampling bias. Normality was tested with the Shapiro–Wilk test. False discovery rate (FDR) correction was carried out for multiple sublineage testing. Finally, the odds ratio between Nextclade clades and categorical variables, at a 95% confidence level, was calculated using binary logistic regression models in SPSS v22.

## 3. Results

### 3.1. Spread and Epidemiology of Delta Sublineages

**Figure 1:**
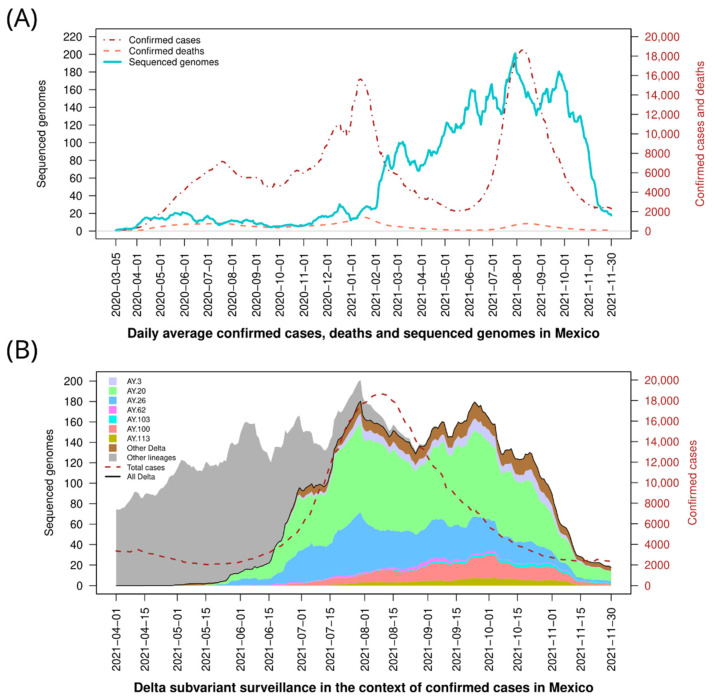
SARS-CoV-2 cases and diversity of Delta variant viruses in Mexico. (**A**) Confirmed cases, deaths, and total genomes sequenced between 1 March 2020, and 30 November 2021 in the country. (**B**) Stacked area plot showing the lineage diversity from April to November 2021. Vertically, lineages are stacked on top of one another. The black line corresponds to the total Delta genomes reported and the dashed red line to the number of confirmed cases for context.

In Mexico, as of March 2022, the pandemic has presented four distinct nationwide epidemiological waves of SARS-CoV-2 (Figure 1A shows the first three waves), with corresponding peaks of cases in June 2020, January 2021, August 2021, and January 2022, respectively. The swift expansion of the Delta VOC ensued during the third epidemiological wave in Mexico, spanning from late June to November 2021 (Figure 1A). Delta overtook the previously prevalent Alpha and Gamma VOCs and the previously dominant lineage B.1.1.519 (Figure S2). Delta showed exponential growth starting in May (5.5%), becoming the most prevalent variant in the country by June (38.6%) and reaching over 97% prevalence in August 2021. Moving forward, our study will focus on Delta genomes from samples collected from May to November 2021.

In Mexico, 89 sublineages of Delta were detected in the analyzed period (Supplementary Table S1), dominated by AY.20, AY.26, and AY.100, which accounted for about 49.4%, 23.7%, and 8.4%, respectively, of all genomes reported in the study period (Figure 1B). Of interest, all three lineages seem to have emerged in Mexico since their prevalence was extremely low in the rest of the world, with a percentage of 0.6%, 1.0%, and 0.7%, respectively (Figure S3). Other less abundant sublineages in the country include AY.3 [Mexico (M): 5.1%; World (W): 3.6%], AY.103 (M: 1.1%; W: 6.4%), AY.113 (M: 2.9%; W: 0.3%), and AY.62 (M: 1.1%; W: 0.1%). These seven Delta sublineages constitute the majority (91.6%; *n* = 18,576) of the cases during Delta circulation in Mexico.

Sublineage AY.20 remained the most prevalent since May, reaching 60% in June and decreasing to 46% in November 2021 (Figure 1B and Figure S2), while AY.26 had a maximum prevalence of 33% during June and dropped to 9% in November. On the other hand, AY.3, AY.100, AY.103, and AY.113 were initially detected at low levels (<1.5%, each) but then increased their prevalence, with a peak in November (7.5%, 12.5%, 3.9%, and 6.3%, respectively) when the Omicron variant reached Mexico. Finally, AY.62 had a very low prevalence nationally, but it was detected almost exclusively in the state of Yucatan in the SE region, with a frequency higher than 10% throughout the evaluated period.

A significant association was found between Delta sublineages and their monthly distribution (at α = 0.05; *p*-value = 0.0001), as well as with the states (*p*-value = 0.0001). On the contrary, no significant associations with cycle threshold values (Ct values), gender, patient status, or age group were observed (at α = 0.05; *p*-values > 0.05 in all cases).

### 3.2. Delta Sublineages Geographical Dispersion

**Figure 2:**
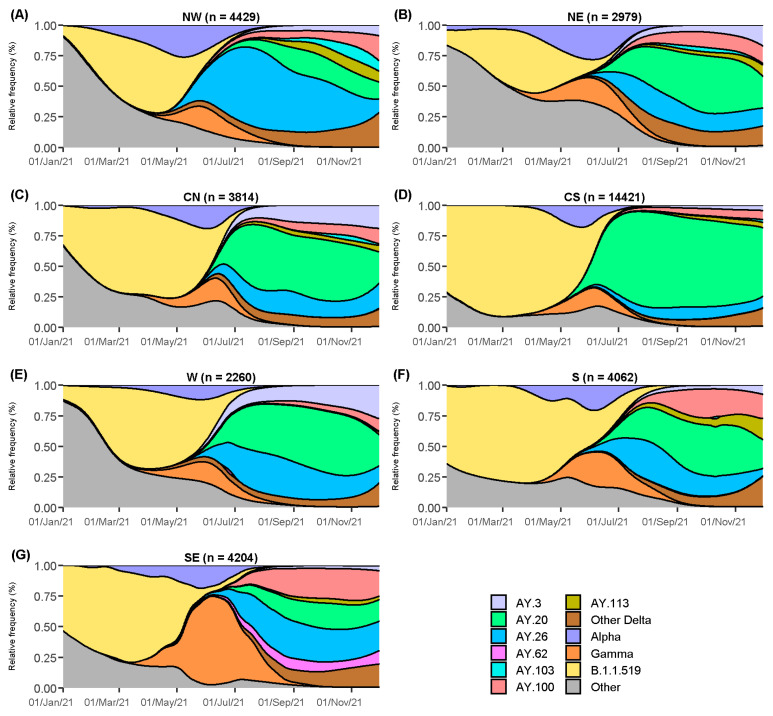
Density plots of relative lineage distribution by geographical region, considering sequences from January to November 2021. (**A**) Northwest, (**B**) Northeast, (**C**) Central North, (**D**) Central South, (**E**) West, (**F**) South, and (**G**) Southeast.

The country was divided into seven geographical regions to analyze the spread of the Delta sublineages throughout Mexico, as seen in Figure S1. Lineage AY.20 was the most prevalent in the country, and Figure 2 shows that it reached at least 75% of all cases in the CS region (Figure 2D); however, it was identified in less than 25% of the cases in the NW (Figure 2A) and SE regions (Figure 2G). Moreover, AY.20 was identified in the CS region as early as May 2021 and spread later to other regions. Contrastingly, the most abundant lineage in the NW was AY.26 (>65%), appearing before May 2021. This lineage was also detected throughout the country, although further ahead in time and at a lower percentage (8–33%). Likewise, AY.103 was present almost exclusively in the NW region and extended to the NE and CN but did not spread further. In the SE, the lineages AY.20, AY.26, and AY.100 circulated with similar frequencies, while the lineage AY.62 was only detected in this region (Figure 2G) suggesting a local transmission chain. In the S region (Figure 2F), the AY.20, AY.26, AY.100, and AY.113 lineages were also frequently identified. Regarding the NE, CN, and W regions, AY.3 (15–25%), AY.20 (45–53%), and AY.26 (19–25%) were the most abundant sublineages (Figure 2B,C,E). In these regions, the Pearson correlation coefficient, calculated between their sublineages’ monthly distributions, was greater than 0.94 (*p* < 0.05), suggesting similar spread patterns of these sublineages over time; thus, they were considered a single region (NE + CN + W) in subsequent analyses.

### 3.3. Mutations in Delta AY.20, AY.26, and AY.100 Sublineages

To further characterize the most prevalent sublineages in Mexico, the defining mutations of AY.20, AY.26, and AY.100 were examined. All available Mexican sequences of these sublineages were analyzed, resulting in 16,085 genomes (Supplementary Table S1). In total, 14,541 nucleotide changes were identified. Of these, 57.8% (*n* = 8429) were nonsynonymous SNPs, whereas the rest were synonymous (*n* = 6112). Among the nonsynonymous SNPs, only 66 showed a prevalence higher than 1%. When analyzing the accumulation of mutation patterns by sublineages, it was seen that AY.20 and AY.100 had, on average, more substitutions (40.6 and 41.6, respectively) than AY.26 (35.1). Interestingly, AY.20 and AY.100 belong to the 21J Nextclade Delta subclade, the most widespread in Mexico, whereas AY.26 falls within subclade 21I which started decreasing before Omicron appeared.

**Figure 3:**
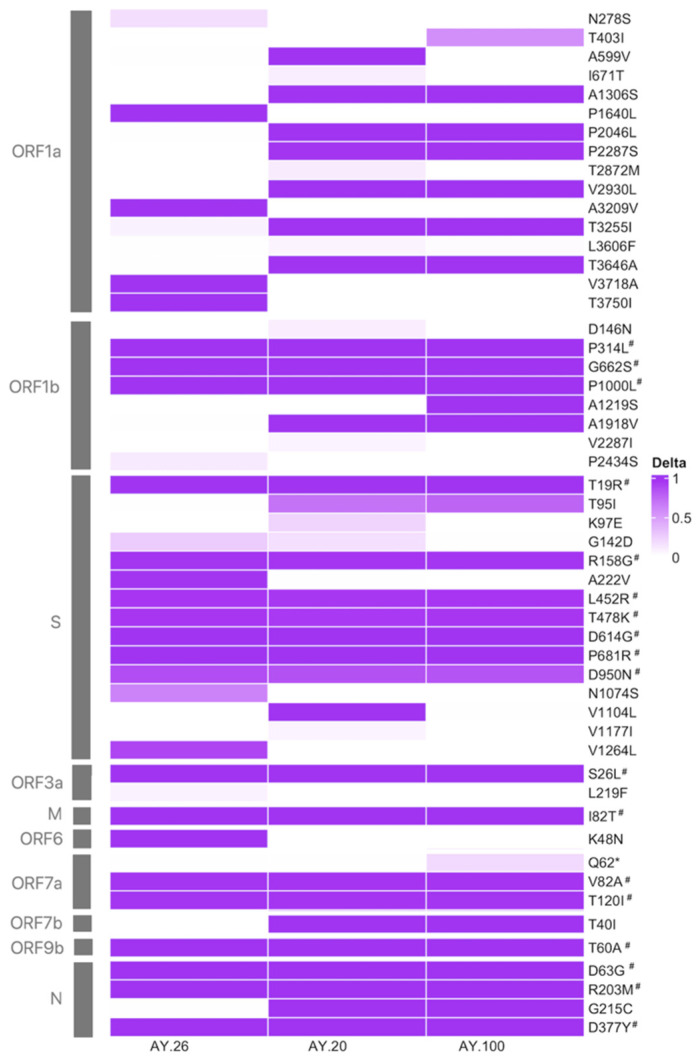
Frequency of aa changes in SARS-CoV-2 genome sequences of Mexico compared with the Wuhan reference genome (NC_045512). Only changes above 5% prevalence in at least one of the sublineages are shown. * Denotes an stop codon. The Delta-defining substitutions are marked with #.

In Figure 3, all mutations with a prevalence higher than 5% in at least one sublineage are shown. The 18 defining changes of the Delta variant (B.1.1.617.2), shared by all its sublineages, can be observed. Seven substitutions are found in spike (S), three in ORF1b and N, two in ORF7a, and one in ORF3a, M, and ORF9b, with none in ORF1a, E, ORF6, ORF7b, nor ORF8. In addition, all Delta sequences harbor two aa deletions (S:157/158 and ORF8:119/120) and three stop codons. The sublineages AY.20, AY.26, and AY.100 harbor 13, 9, and 13 additional nonsynonymous changes, respectively, compared to the signature mutations of the Delta variant (Figure 3).

Some sublineage-specific substitutions were present in prevalences ranging from 86% to 99% of their analyzed genomic sequences; eight in lineage AY.26 (four in ORF1a, three in S, and one in ORF6, see Figure 3), two were only present in AY.20 (ORF1a:A599V and S:V1104L), and one in AY.100 (ORF1b:A1219S). Additionally, AY.20 and AY.100, which are placed in clade 21J of the Nextclade classification, also share ten high-frequency substitutions (six in ORF1a, and one in each ORF1b, S, ORF7b, and N). Moreover, other substitutions had a prevalence of <50%, suggesting that these three sublineages may have distinct local transmission chains that led to local viral subpopulations. This observation was further explored using a haplotype network and a phylogenetic reconstruction.

### 3.4. Delta Haplotype Network

**Figure 4:**
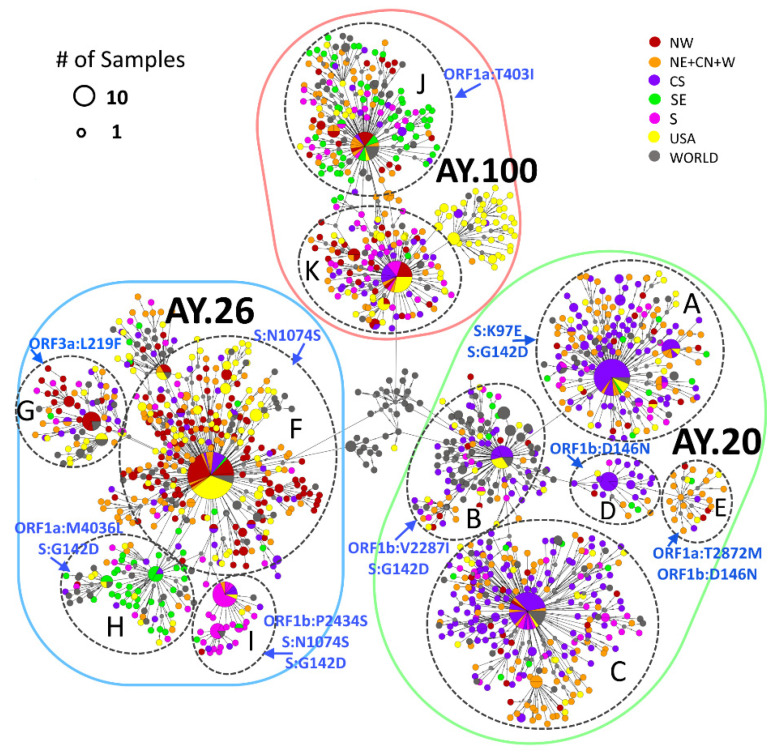
Haplotype network using mutations from AY.20, AY.26, and AY.100 sublineages in Mexico and worldwide. Colors indicate the Mexican regions sequences were isolated, with yellow and gray circles representing the USA and the rest of the world, respectively. The size of the circles indicates the number of samples within the same haplotype (scale is provided). Main subclusters within each lineage are marked with black discontinuous circles, and their specific mutations are indicated in blue.

A time-scaled phylogenetic tree and a haplotype network were built to further explore the spread of Delta sublineages at Mexico’s regional level. The NE, CN, and W regions (Figure S1) were collated into a single region (NE + CN + W) given the high correlation of circulating sublineages in these areas over time. The network in Figure 4 reveals a clear separation of the sublineages and their association with specific regions in which they circulated; for lineage AY.20, three large (marked as A, B, and C) and two minor (D and E) subclusters can be clearly identified, indicating that different haplotypes of AY.20 may have circulated together. In the periphery of the main haplotypes (corresponding to the largest circles) of the A and C subclusters, many sequences from the CS (purple) region were present, but also from the NE + CN + W (orange) region. Correspondingly, in the phylogeny (Figure S4), AY.20 sequences from the CS region appeared earlier than those from the NE + CN + W, suggesting that the spread of this sublineage started there.

**Figure 5:**
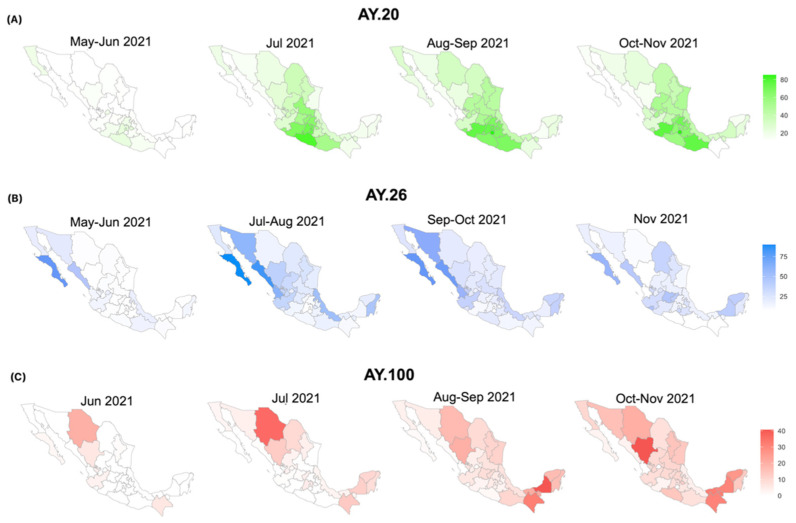
Map series showing the spatiotemporal distribution of the most prevalent Delta sublineages in Mexico. (**A**) Sublineage AY.20, (**B**) sublineage AY.26, (**C**) sublineage AY.100. The prevalence was calculated for each state over time.

Interestingly, sequences within subcluster B, which had the highest number of international samples (grey), harbor two additional nonsynonymous substitutions (ORF1b:V2287I and S:G142D) (Figure 4). This contrasts with subcluster C, comprised mainly of Mexican sequences, which carry only sublineage-specific mutations. These observations also support the idea that the original AY.20 started its circulation in the CS region and acquired additional mutations as it spread. The spatiotemporal distribution of AY.20 (Figure 5A) shows that the earliest identification was observed in the CS region, specifically in Mexico City (28% in May–June), and in the South region with the highest prevalence in Guerrero (14% in May–June), following dispersion to other regions, especially the NE + CN + W.

For the sublineage AY.26, the network revealed the presence of different haplotypes with distinct geographical distributions. The largest subcluster F, as well as G, were dominated by sequences obtained from samples collected in the NW region (red), NE + CN + W regions (orange), and USA (yellow). Additionally, subcluster I from the SE region (green) and H from the SE region (green) derived from F, suggesting different haplotypes with local transmission chains. These observations were consistent with the phylogeny (Figure S4), where samples from the NW region dominated, especially at earlier dates, along with a few sequences from the SE region. Later, sequences from NE + CN + W regions appeared, followed by sequences from the rest of the country, but with distinct clades for the S and SE regions. Moreover, the data shown in Figure 5B confirms that AY.26 spread initially in the northern states of Mexico, probably corresponding to haplotypes F and G, and at a lower frequency in the SE region with haplotypes H and I; then, this lineage spread to the NE + CN + W and S regions.

Finally, the sublineage AY.100 had two main subclusters: J and K (Figure 4). Subcluster K included sequences mainly from the USA (yellow) and all regions of Mexico, except the SE region (green). In subcluster J, samples were from the SE region (green) and at a lower prevalence than the rest of the country, suggesting two separate transmission chains for these haplotypes: one that started spreading from the North, and another from the South. These patterns matched those observed in the phylogeny (Figure S4) and the spatiotemporal distribution of AY.100 (Figure 5C).

Of note, some of the less-frequent mutations (present in <85% of the total Mexican Delta sequences) found in the heatmap (Figure 3) correspond to a particular subcluster in the network, supporting the idea of different local transmission chains of Delta sublineages. For instance, the mutation S:K97E, present in only 21.6% of AY.20 genomes, was present in subcluster A (Figure 4). Additionally, the mutation ORF1a:T403I, identified in 55.7% of AY.100 sequences, was only found in subcluster J. On the other hand, some less-frequent mutations, such as S:G142D, were shared among sublineages. This mutation was present in 25.8% of AY.20 sequences corresponding to subclusters A and B, and in 9.8% of AY.26 sequences belonging to subclusters H and I. In some cases, the subclusters with additional mutations were restricted to a geographical region, as can be observed in subclusters H and I of AY.26, which share mutations S:G142D (25.8% prevalence) and S:N1074S (61.4% prevalence), plus ORF1b:P2434S (10.2% prevalence) in H, and ORF1a:N278S (16.4% prevalence) in I.

## 4. Discussion

The Delta variant of SARS-CoV-2 spread worldwide during 2021 and replaced most previously circulating lineages due to its transmission advantage and its ability to partially evade pre-existing immunity [30]. The ample circulation of Delta led to a regional diversification that resulted in 133 sublineages. In Mexico, Delta was principally responsible for the third wave of the COVID-19 pandemic, accounting for more than 99% of infections by November 2021.

In contrast to other countries in which the Delta variant was associated with an increased severity of COVID-19 in unvaccinated patients (higher numbers of hospitalizations and severe symptoms) [31,32], in Mexico, the proportion of severe illness and case-fatality rates remained lower than in the previous waves [12]. Despite having low vaccination rates when the first cases of Delta were detected in Mexico (<9% with a complete initial vaccination protocol and <12% partly vaccinated) and during its peak surge (<43% and <24%, respectively) [33], hospitalization and mortality decreased in Mexico in comparison to the second wave. Various factors may have contributed to lessening the impact of the Delta wave, such as the decision of health authorities to prioritize the vaccination of older people, a better knowledge of the medical treatment of the disease, and an estimated seroprevalence of 23.4% before the entry of Delta [34,35]. However, by the end of the third wave, seroprevalence had reached 86.7%, probably due to the onset of Delta and the progress in vaccination (Muñoz-Medina JE et al., in preparation).

This work describes the presence and geographical distribution of the Delta sublineages AY.3, AY.20, AY.26, AY.62, AY.100, and AY.113, which were the most prevalent in Mexico, with a particular focus on the three most abundant (AY.20, AY.26, and AY.100). It seems that migration patterns could have influenced the sublineage distribution chains. For instance, the sublineages AY.100 and AY.113 were highly prevalent in the S and SE regions, correlating with their maximum frequencies in Central American countries such as Costa Rica (3.4 and 21%, respectively) and Guatemala (30 and 68%). This observation may reflect the continuous influx of migrants from Central America to Mexico. However, AY.100 was also detected early in the NW, suggesting at least two possible spread chains of distinct haplotypes in each region. By contrast, the sublineage AY.3, which was equally prevalent in the USA and Mexico, exhibited a higher prevalence only in the NE + CN + W regions (15–25%). A very interesting case was sublineage AY.62, which, in Mexico, was almost exclusively found in the state of Yucatan in the SE region, where it reached 20% prevalence from July to September. This lineage was also detected in the USA, particularly in Texas and Kentucky; however, it only accounted for 0.75% of genomes in those locations during the period, suggesting that it may have entered Mexico through the tourism hotspots within the region and stayed there.

Interestingly, the sublineages AY.20 and AY.26 seem to be almost exclusive to Mexico, representing nearly 74% of Delta infections in the country, while it was detected at a much lower prevalence (<1.6%) elsewhere (Figure S3). Regarding the sublineage AY.20, all subclusters in the network were dominated by samples from the CS region, particularly Mexico City, which also corresponded to the earliest samples in the phylogeny and on the map (Figure 5 and Figure S4). Therefore, this sublineage either entered or originated in Mexico City and later spread efficiently towards other regions. The haplotype network and mutation analysis showed limited regional clustering of AY.20 populations, but different spread chains could be observed. Interestingly, the AY.26 sublineage was detected early in the north of Mexico, particularly in the NW region, although the bordering USA states did not exhibit high frequencies of this sublineage (California and Arizona reported a frequency of AY.26 of 5% vs. 50% in NW Mexico, according to GISAID data), indicating that the exchange of AY.26 through the Mexico–US border was limited in contrast to what has been reported for the Alpha variant [14]. This data suggests that this sublineage originated in the NW of Mexico. Interestingly, the prevalence of AY.26 decreased by November 2021 (<10%), as did the majority of worldwide Delta sublineages belonging to clade 21I (of Nextclade), while AY.20 remained the most prevalent (>44%) until the expansion of the Omicron variant. Overall, AY.20 started to spread from central and southern Mexico, where it reached its highest prevalence, whereas AY.26 and AY.100 seem to have spread simultaneously from the NW and the S/SE regions towards the center of the country and the NE + CN + W regions. In general, the region NE + CN + W seems to be a transition area where the three main sublineages co-circulated but were introduced at different timepoints.

Mutation analysis was carried out for the three most prevalent sublineages: AY.20, AY.26, and AY.100. Notably, in addition to the defining Delta substitutions in the spike protein, namely L452R, T478K, D614G, and P681R, which increase transmissibility and immune escape [5,6,7,30], other substitutions were identified within these sublineages which may have also played a role in enhanced Delta phenotypes. For instance, several substitutions in the N-terminal domain of S were present, including T19R and R158G, which were shared among the three sublineages: T95I was shared by AY.20 and AY.100; and G142D was shared by AY.20 and AY.26. These substitutions have been identified in other Delta sublineages such as AY.4 and some of them in Omicron (T95I and G142D). The role of each specific mutation has not been completely elucidated, but they have all been implicated in immune escape [36,37,38]. In addition, the mutation S:A222V, present in AY.26, slightly increases human cell receptor (ACE2) binding [39], while the mutations N:R203M, common to the three sublineages, and N:G215C, present in AY.20 and AY.100, have been shown to improve viral replication and assembly [40,41]. Even though Delta-defining mutations do not include changes in ORF1a, many Delta sublineages harbor specific mutations in this ORF. Here, we identified 16 aa changes in ORF1a, 6 of which were shared by AY.20 and AY.100, probably due to their placement within the Nextclade subclade 21J. Despite the abundance of mutations in ORF1ab in all SARS-CoV-2 genomes, most remain uncharacterized.

## 5. Conclusions

This study describes the evolution of the most prevalent sublineages of the Delta variant in Mexico, contributing to a better understanding of their dynamics and spread. This knowledge is relevant since new SARS-CoV-2 lineages and mutations have the potential to substantially impact public health systems as they can potentially lead to an increase in infectivity, transmissibility, and/or disease severity. Additionally, the description of the circulation dynamics of SARS-CoV-2 within the country will aid public health authorities in focusing their genomic surveillance efforts in the states or regions where newly introduced variants are repeatedly detected, as the NW and SE regions and Mexico City.

## Data Availability

The generated sequences of SARS-CoV-2 used in this study are publicly available through the Global Initiative on Sharing All Influenza Data (GISAID) repository and CoViGen genomes have also been deposited in the Genbank NCBI database. Accession numbers are listed in Supplementary Table S1.

## Supplementary Materials

The following supporting information can be downloaded at: https://www.mdpi.com/article/10.3390/v14061165/s1, Figure S1: Map of Mexico with the seven geographical regions indicated; Figure S2: Stacked density plot of relative frequency of SARS-CoV-2 lineage circulation in Mexico; Figure S3: Frequency of the most prevalent Delta sublineages in Mexico; Figure S4: Time-scaled, maximum likelihood phylogeny; Table S1: Patient demographic and clinical characteristics of Mexican sequences classified as Delta and reported as of 14 December 2021.
