# Supplementary material for: Dominance of Three Sublineages of the SARS-CoV-2 Delta Variant in Mexico
Source: Viruses. 2022 May 27;14(6):1165. doi: 10.3390/v14061165 (PMC9229647; doi:10.3390/v14061165)
Supplement: Supplementary file 1 [file viruses-14-01165-s001.zip › SuppFigures_v4.pdf]

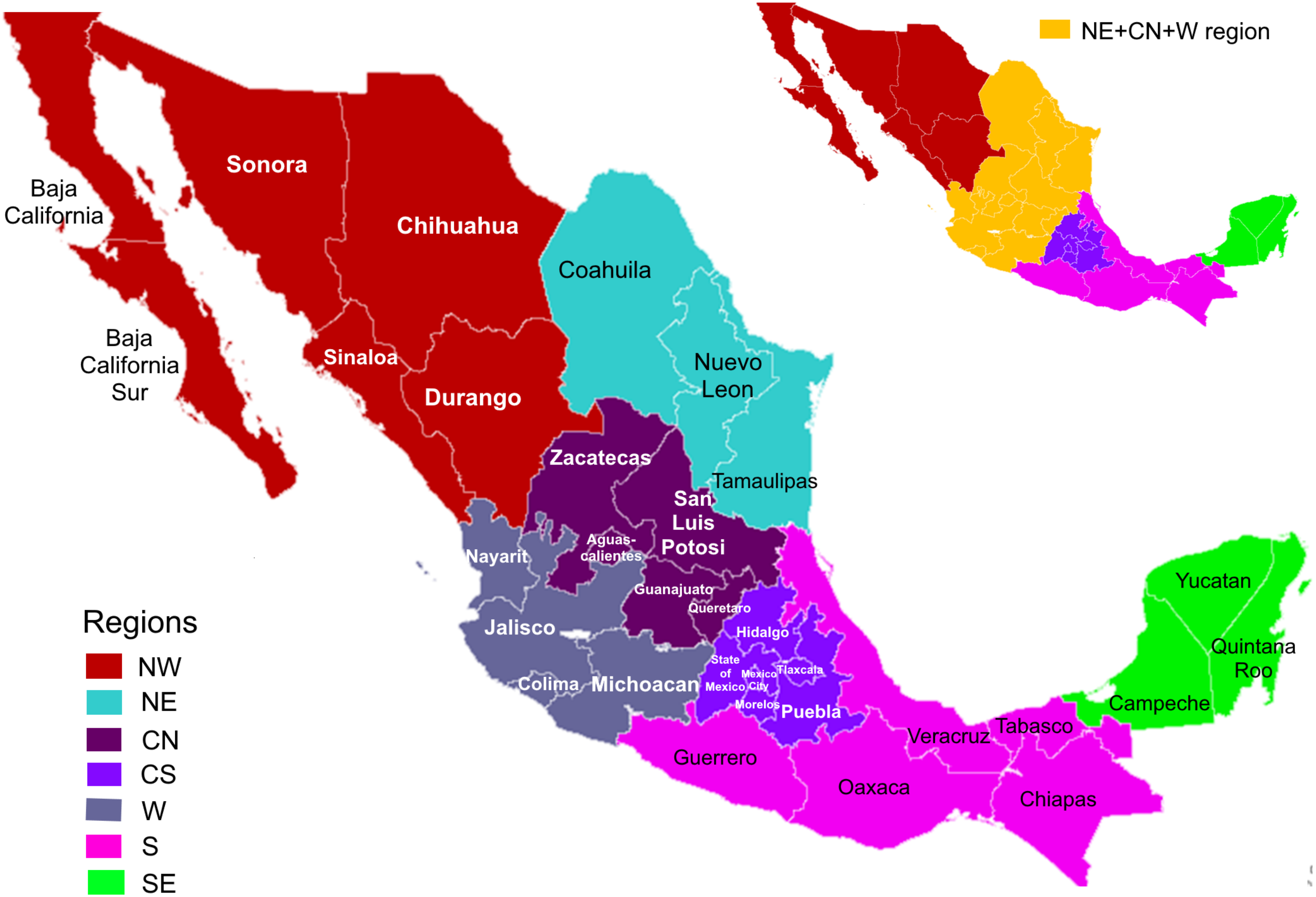

Figure S1. Map of Mexico with the seven geographical regions indicated

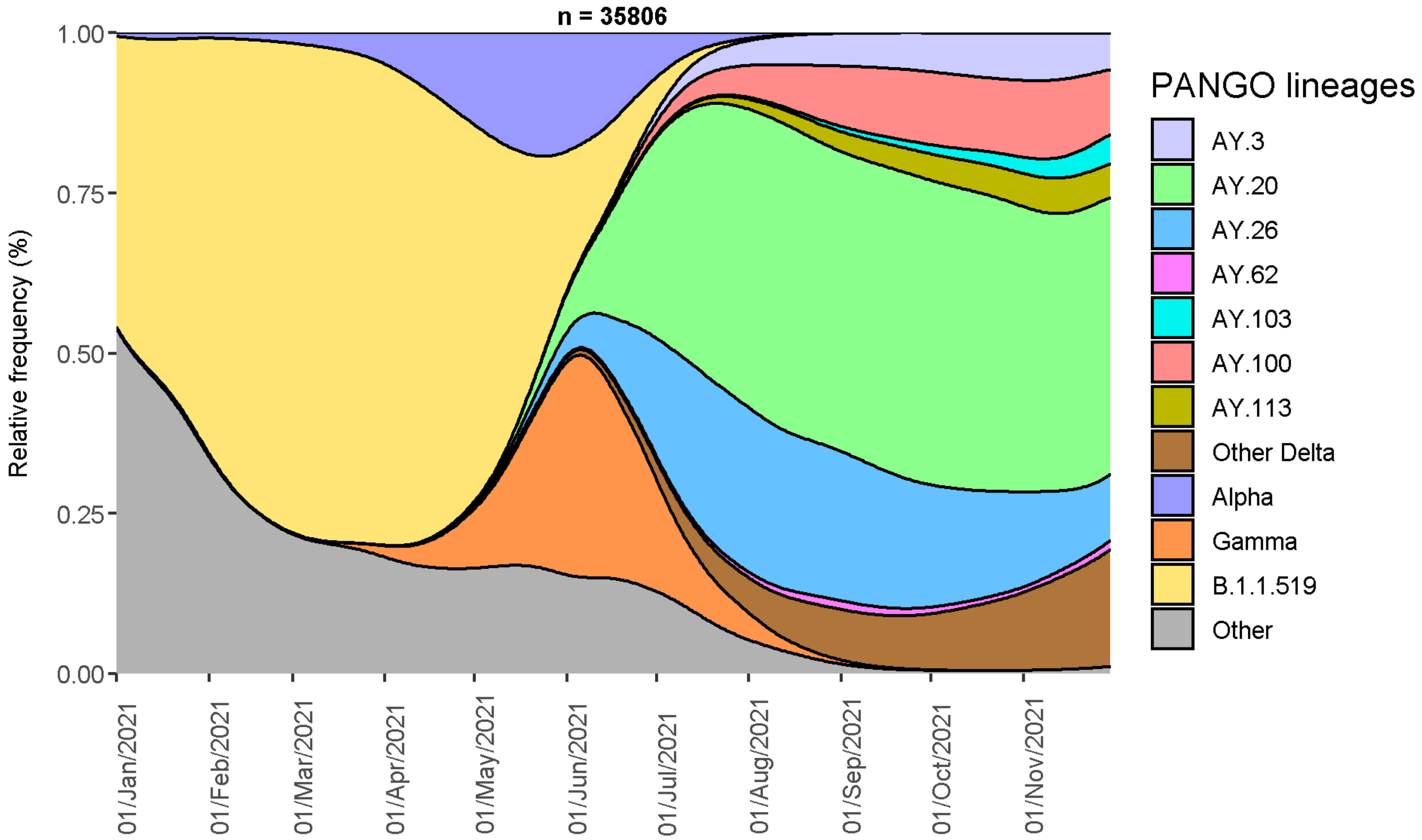

Figure S2. Stacked density plot of relative frequency of SARS-CoV-2 lineage circulation in Mexico

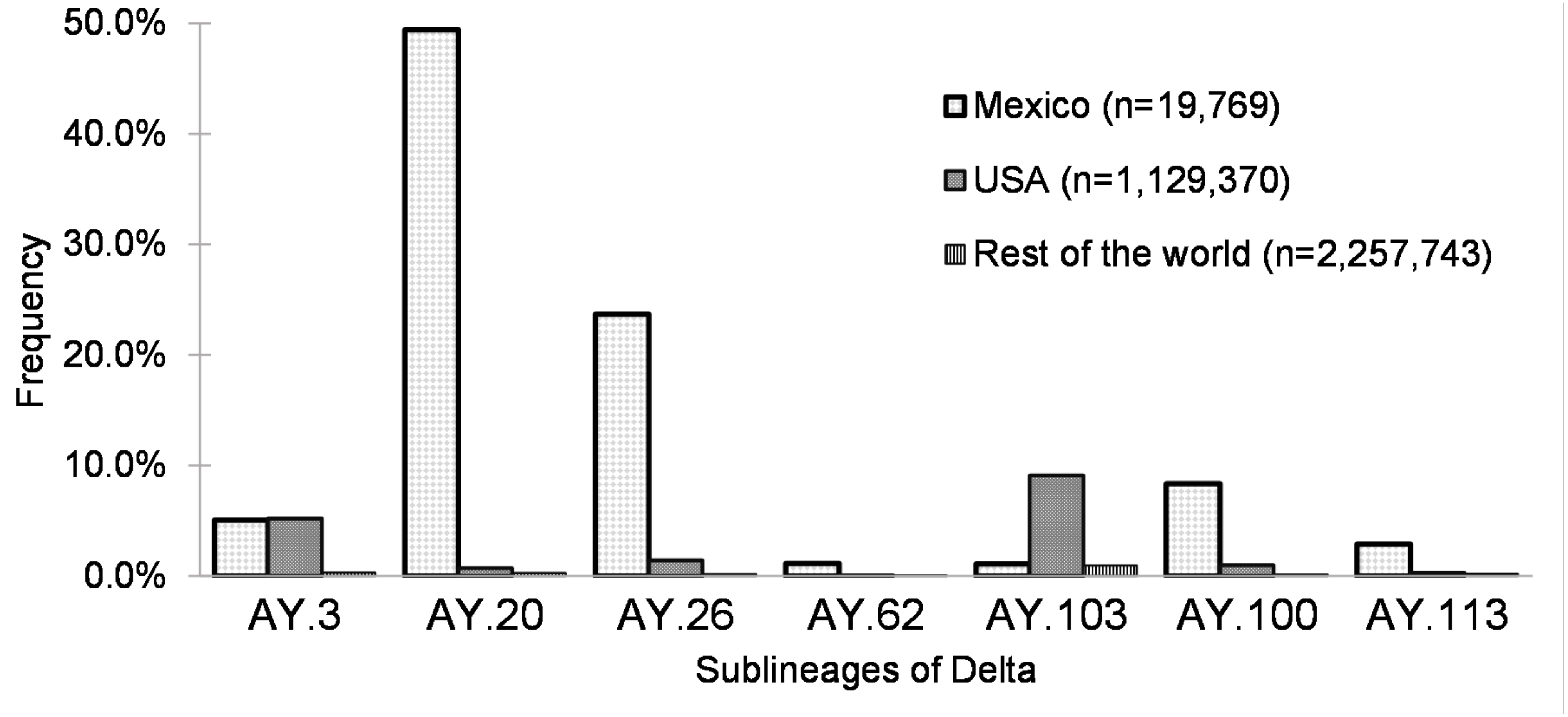

Figure S3. Frequency of the most prevalent Delta sublineages in Mexico. Sublineage sampling frequency in Mexico, the USA, and the rest of the world is shown.

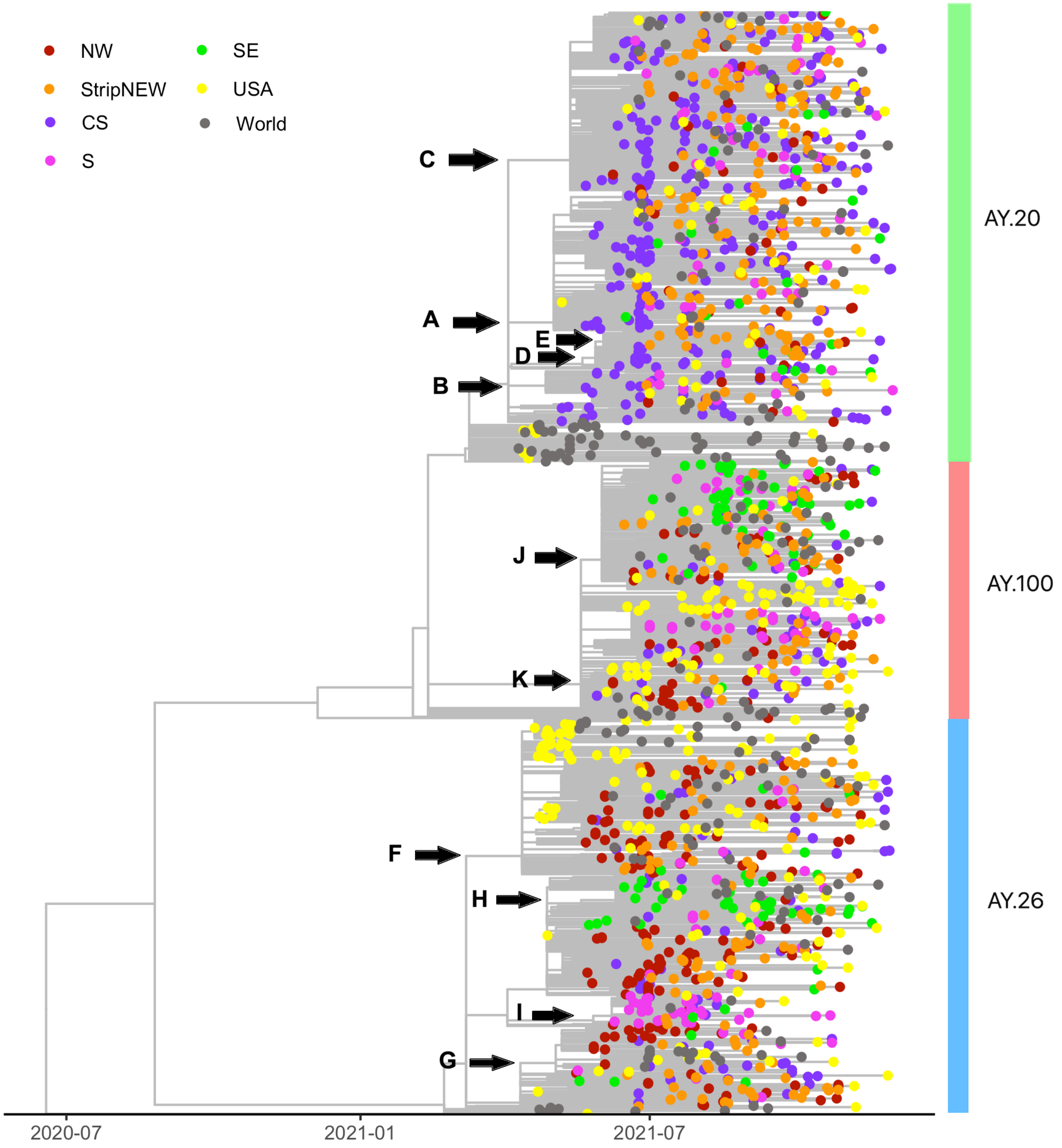

Figure S4. Time-scaled, maximum likelihood phylogeny. The tips are colored by the region of sampling and the sublineage is indicated at the right. Haplotype clusters' location is indicated by black arrows.
